# High prevalence of *Schistosoma mansoni* and other intestinal parasites among elementary school children in Southwest Ethiopia: a cross-sectional study

**DOI:** 10.1186/s12889-015-1952-6

**Published:** 2015-07-02

**Authors:** Ayalew Jejaw, Endalew Zemene, Yayehirad Alemu, Zemenu Mengistie

**Affiliations:** Department of Biomedical Science, College of Health Sciences, Mizan-Tepi University, Mizan, Ethiopia; Department of Medical Laboratory Sciences and Pathology, College of Health Sciences, Jimma University, Jimma, Ethiopia

**Keywords:** *S. mansoni*, Intestinal parasites, School children, Ethiopia

## Abstract

**Background:**

Intestinal parasitic infections (IPIs) pose significant public health challenges in school children in developing countries. The aim of this study is to determine prevalence of intestinal parasites among elementary school children in Mizan-Aman town, southwest Ethiopia.

**Methods:**

Institution-based cross-sectional study involving 460 elementary school children in Mizan-Aman Town was conducted from May to June 2013. The school children were selected using multistage sampling technique. Data on demography and predisposing factors of IPIs were collected using pretested questionnaire. Moreover, single stool specimen was examined microscopically after wet mount and formol-ether sedimentation concentration procedures. Infection intensity of *Schistosoma mansoni* and soil-transmitted helminths (STHs) was estimated using Kato-Katz egg counting method.

**Results:**

Age of the children ranged from 5 to 17 years. Overall, 76.7 % (95%CI: 72.8–80.6) of the children harbored at least one species of intestinal parasite. Eight species of intestinal parasites were detected with *S. mansoni* (44.8 %) and *Ascaris lumbricoides* (28.7 %) being predominant. Helminths and pathogenic intestinal protozoa were detected in 73.9 and 7.8 % of the children, respectively. After adjusting for other variables, age between 5 and 9 years (AOR, 2.6, 95%CI, 1.552–4.298), male gender (AOR, 2.1, 95%CI, 1.222–3.526), attending public school (AOR, 0.1, 95%CI, 0.060–0.256), using river/well water (AOR, 2.4, 95%CI, 0.912–6.191), irregular washing of hands before meal (AOR, 0.5, 95%CI, 0.254–0.865), consuming street food (AOR, 2.3, 95%CI, 1.341–3.813) and raw vegetables (AOR, 2.7, 95%CI, 1.594–4.540) were significantly associated with IPIs in the study participants.

**Conclusion:**

Prevalence of intestinal parasites among the school children was high. Deworming of the school children and continuous follow up is required.

## Background

Intestinal parasitic infections (IPIs) are the most common infections in developing countries, where sanitary facilities are scarce. Globally, the three most common soil-transmitted helminths (STHs) *Ascaris lumbricoides*, *Trichuris trichiura* and the anthropophilic hookworms (*Ancylostoma duodenale* and *Necator americanus*) are responsible for majority of the disease burden due to neglected tropical diseases. It is estimated that 819.0, 464.6 and 438.9 million people are infected with *A. lumbricoides, T. trichiura* and the hookworms, respectively [1]. Schistosomes affect an estimated 240 million people worldwide, with the risk in Africa commonly associated with development of water resources [2]. While the parasites may infect people of all ages in impoverished communities, children are more likely to get infected.

Young children and individuals with heavy worm burden suffer the most from morbidity associated with the STH and *S. mansoni* infections. Although mortality due to these infections is rare, the morbidity and detrimental effects on socio-economic development resulting from these infections is enormous [3–7]. Apart from the direct health impacts, malaria-helminth co-infections may also increase severity of malarial anemia resulting in gametocyte carriage, perhaps affecting transmission dynamics of malaria in endemic areas [8].

Deworming of helminth infected children is required to reduce the nutritional deficit resulting from these infections [9, 10]. The World Health Organization (WHO) recommends mass school-based deworming of school-age children twice a year if prevalence of the STHs ≥ 50 %, and once every year if the prevalence is ≥ 20 % and less than 50 % [11].

Baseline surveys of IPIs produce essential evidence to take appropriate interventions, particularly for control of STHs and *Schistosoma* infections. Most of the epidemiological studies of intestinal parasites carried out in Ethiopia documented often high prevalence of intestinal parasites among school children, with the STHs being most predominant [12, 13]. School-age children in developing countries, including Ethiopia, are typically at increased risk of IPIs as a result prevailing predisposing factors [14, 15]. Despite these, no published report on the magnitude of IPIs among elementary school children in Mizan-Aman Town was obtained. This research is, therefore, initiated with the objective of determining prevalence of intestinal parasites among school children enrolled in elementary schools in the town.

## Methods

### Study setting

**Fig. 1:**
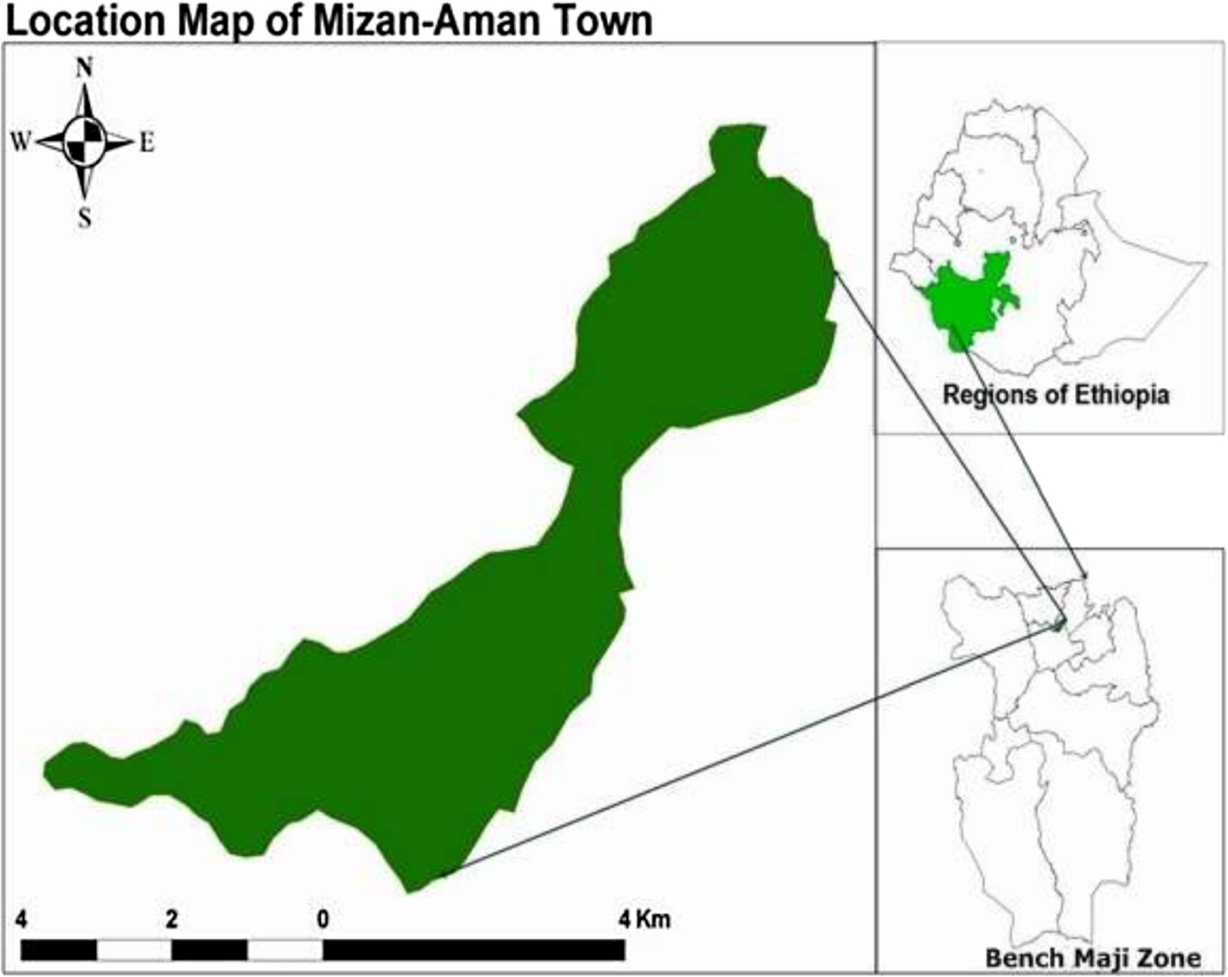
Map of the study area

The study was conducted in Mizan-Aman Town from May to June, 2013. Mizan-Aman Town is found in Bench Maji Zone of the Southern Nations, Nationalities and Peoples’ Region of Ethiopia (Fig. 1). The town is located 550 kms southwest of the capital Addis Ababa. The area is characterized by warm climate (mean annual temperature ranges between 15.1 and 27 °C), perennial rivers, and is considered ideal for agriculture and human settlement. The mean annual rainfall ranges from 400 to 2000 mm. The major economic activity of the urban inhabitants is trading while subsistence farming is the dominant means of earning a living for the surrounding rural population. In the year 2013, a total of 18 elementary schools (11 public and 7 private) were present in the town. A total of 14,393 children were enrolled in the elementary schools in the town in the year 2014.

### Study design and sample size determination

School-based cross-sectional study was conducted. Sample size was determined using single population proportion formula *n* = Z^2^ p (1-p) / d^2^, with the following assumptions: prevalence (p) of 83.8 % from a previous study [16], 95 % confidence level, 5 % margin of error, design effect of 2 and 10 % for anticipated non-response rate. Accordingly, the minimum sample size (n) was found to be 460 school children.

Multistage sampling was employed to select the study participants. First, seven of the elementary schools were randomly selected by lottery method. The sample size was allocated to the selected elementary schools proportional to the total number of student population in each of the schools. Accordingly, 145, 144, 63, 60, 31, 10 and 7 school children were sampled from Mizan Number One, Aman, Ediget Behibret, Gacheb, Mizan Misgana Academy, Aman Misgana Academy and Abune Teklehaymanot elementary schools, respectively. The school children were selected by systematic sampling technique, using list of the students as sampling frame. Children attending school in the selected elementary schools during the study period, who were voluntary to participate in the study and able to provide stool sample within the study period were included in this study.

### Demographic and personal risk factors survey

Data on demographic profile of the children and factors predisposing to IPIs was gathered using pretested questionnaire. The questions included information on gender, age, shoe wearing pattern, source of drinking water, bathing in the river, habit of eating raw vegetables and street foods among others. Trained nurses who were fluent with the local languages (*Amharic* and *Bench*) interviewed the study participants.

### Parasitological examination

Single stool specimen was collected from each study participant using clean plastic container labeled with unique identification number. The children were instructed on how to provide at least a thumb-sized stool specimen of their own, avoiding contamination with urine. The specimens were collected at each school and immediately transported to Clinical Laboratory of Mizan-Tepi University for processing. Direct wet smear using physiological saline and Lugol’s iodine was done. Portion of the stool samples were processed by formol-ether sedimentation concentration technique, and examined microscopically following standard procedure [17]. Moreover, infection intensity of STHs and *S. mansoni* was determined using Kato-Katz technique. A single Kato-Katz slide was processed for each stool specimen. The specimens were processed on the same day of collection. Results of the laboratory investigation were recorded on a format prepared for this purpose.

### Data analysis

Infection intensity of the STHs and *S. mansoni* was estimated by multiplying the total number of eggs counted by 24, which gives as the eggs per gram (epg) of stool. Infection intensities of *S. mansoni* and the STHs were classified as light, moderate and heavy per the threshold set by WHO [18, 19]. The collected data were checked for completeness, entered into computer, and analyzed using SPSS version 20.0 software package. Descriptive statistics were used to summarize demographic profile of the study participants. Bivariate and multivariable logistic regression procedures were employed to identify factors associated with IPIs in the study participants. Variables appearing significantly associated with IPI by the bivariate analysis, and other biologically plausible variables were candidates for the multivariable model. The multivariable model was fitted by backward elimination technique. Statistical significance was set at p value < 0.05.

### Ethical clearance

Ethical clearance was obtained from the Institute of Research and Community Support of Mizan-Tepi University. Permission was sought from Mizan-Aman Town Education and Health Offices. Informed verbal consent was obtained from the students and directors of each school. Parent/guardian consent was obtained for minors to participate in the study. Laboratory results were kept confidential. Students with positive results for intestinal parasite(s) were treated in collaboration with the health centres in the town.

## Results

### Characteristics of the study participants

Table 1 shows demographic characteristics of the study participants. A total of 460 school children (50.4 % female and 49.6 % male) participated in the study. Age of the children ranged from 5 to 17 years (mean age 9.1 years). Most of the children (64.1 %) were within the age group 5–9 years.

**Table 1:** Demographic characteristics and intestinal parasitic infection among the school children, Mizan-Aman Town, 2013

Demographic characteristics	Intestinal parasite		Total
	Positive n(%)	Negative n(%)	
Gender			
Male	190(83.3)	38(16.7)	228(49.6)
Female	163(70.3)	69(29.7)	232(50.4)
Age group (years)			
5–9	242 (82.0)	53 (18.0)	295 (64.1)
≥ 10	111 (67.3)	54 (32.7)	165 (35.9)
Type of school			
Public	336 (81.6)	76 (18.4)	412 (89.6)
Private	17 (35.4)	31 (64.6)	48 (10.4)

### Prevalence of intestinal parasites and associated risk factors

**Fig. 2:**
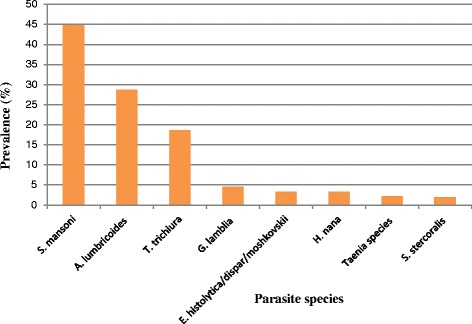
Species distribution of intestinal parasites detected among the elementary school children in Mizan-Aman Town, 20013

At least one species of intestinal parasite was detected in 76.7 % (95%CI: 72.8–80.6) of the children. Eight species of intestinal parasites were identified. *S. mansoni* was the most common intestinal parasite detected (44.8 %), followed by *A. lumbricoides* (28.7 %). Prevalence of each intestinal parasite is demonstrated in Fig. 2.

Out of the total children, 340 (73.9 %) were positive for at least one species of intestinal helminth, and 36 (7.8 %) were positive for protozoan parasites (*Giardia lamblia* and *Entamoeba histolytica/dispar/moshkovskii*). Risk factors associated with helminth infections include consumption of undercooked/raw vegetables (AOR 2.8, 95%CI, 1.705–4.889), consumption of street food (AOR 2.5, 95%CI, 1.523–4.368), male gender (AOR, 2.3, 95%CI, 1.392–4.043), age less than ten years (AOR 3.2, 95%CI, 1.880–5.498), being enrolled in public elementary school (AOR 0.095, 95%CI, 0.043–0.212), using river water for drinking (AOR 4.385, 95%CI, 2.504–7.679). Two hundred and sixty nine (58.5 %) of the children responded to bath and wash clothes in the river. Prevalence of *S. mansoni* among these children (64 %) was significantly higher than those who responded not taking bath and washing clothes in the river (data not presented).

Prevalence of intestinal parasites among the school children in each school included in the study is presented in Table 2. There was a significant difference in prevalence of intestinal parasites in the children among the schools (*p* < 0.001). Moreover, prevalence of intestinal parasites among the school children enrolled in public elementary schools (84.1 %) was significantly higher than those enrolled in private schools (35.4 %) (*p* < 0.001). Multiple IPIs were detected in 35.4 % of the infected children. *S. mansoni*/*A. lumbricoides* double infections (47 %) and *S. mansoni/A. lumbricoides*/*T. trichiura* triple infections (34 %) were the most commonly encountered multiple infections.

**Table 2:** Prevalence of intestinal parasites detected among the school children in each school, Mizan-Aman Town, 2013

Name of the elementary school	Number of children included	Prevalence of intestinal parasite n(%)	95% CI
Gacheb	60	54 (90.0)	(82.4–97.6)
Ediget Behibret	63	56 (88.9)	(81.0–96.7)
Mizan Number One	145	121 (83.4)	(77.3–89.5)
Aman	144	105 (72.9)	(65.6–80.2)
Abune Teklehaymanot^a^	7	3 (42.9)	(6.2–79.6)
Mizan Misgana Academy^a^	31	11 (35.5)	(18.7–52.3)
Aman Misgana Academy^a^	10	3 (30.0)	(1.6–58.4)
Total	460	353 (76.7)	(72.8–80.6)

*CI* Confidence interval

^a^Private schools

Infection intensity of *S. mansoni* and the STHs detected in this study is shown in Table 3. Most of the *S. mansoni* and *T. trichiura* infected children had moderate infection intensity. Heavy infection with *S. mansoni, A. lumbricoides* and *T. trichiura* was obtained 4.4, 5.3 and 3.5 % of the children infected with each of the parasites, respectively.

**Table 3:** Infection intensity of *S. mansoni* and the STHs detected among the school children in Mizan-Aman Town, 2013

Parasite species	Infection intensity			
	Light n(%)	Moderate n(%)	Heavy n(%)	Mean egg count (epg)
*S. mansoni* (*n =* 206)	72 (35)	125 (60.7)	9 (4.4)	175 ± 107
*A. lumbricoides* (*n =* 132)	76 (57.6)	49 (37.1)	7 (5.3)	15,263 ± 1,9857
*T. trichiura* (*n =* 86)	32 (37.2)	51 (59.3)	3 (3.5)	3295 ± 4163

*epg* eggs per gram

Independent risk factors associated with IPI among the school children is presented in Table 4. After adjusting for other variables, male gender (AOR 2.1, 95%CI, 1.222–3.526), age group within 5–9 years (AOR 2.6, 95%CI, 1.552–4.298), attending public school (AOR 0.1, 95%CI, 0.060–0.256), using well/river as source of water supply (AOR 2.4, 95%CI, 0.912–6.191), irregular washing of hands before meal (AOR 0.5, 95%CI, 0.254–0.865), habit of eating raw vegetables (AOR 2.7, 95%CI, 1.594–4.540) and consumption of street foods (AOR 2.3, 95%CI, 1.341–3.813) were significantly associated with IPI among the study participants.

**Table 4:** Predicators of intestinal parasitic infections among the school children (*n =* 460) in Mizan-Aman Town, 2013

Variables		Intestinal parasite		COR (95 % CI)	AOR (95 % CI)
		Positive n(%)	Negative n(%)		
Age group in years	5–9	242 (82.0)	53 (18.0)	2.2 (1.430–3.451)^*^	2.6 (1.552–4.298)^*^
	≥10	111 (67.3)	54 (32.7)	1	1
Gender	Male	190 (83.3)	38 (16.7)	2.1 (1.353–3.312)^*^	2.1 (1.222–3.526)^*^
	Female	163 (70.2)	69 (29.8)	1	1
Type of school	Public	336 (81.6)	76 (18.4)	0.1 (0.065–0.236)^*^	0.1 (0.060–0.256)^*^
	Private	17 (35.4)	31 (64.6)	1	1
Source of water	Well/river	54 (90.0)	6 (10)	3.0 (1.270–7.279) *	2.4 (0.912–6.191)*
	Tap	299 (74.8)	101 (25.2)	1	1
Eating raw vegetables	Yes	196 (85.6)	33 (14.4)	2.8 (1.766–4.438)*	2.7 (1.594–4.540)*
	No	157 (68)	74 (32)	1	1
Consumption of street foods	Yes	224 (82.1)	49 (17.9)	2.1 (1.327–3.184)^*^	2.3 (1.341–3.813)^*^
	No	129 (69)	58 (31)	1	1
Latrine availability at home	Yes	319 (77.2)	94 (22.8)	1	1
	No	34 (72.3)	13 (27.7)	0.7 (0.391–1.520)	1.3 (0.548–3.010)
Hand washing before meal	Always	229 (72.5)	87 (27.5)	1	1
	Sometimes	124 (86.1)	20 (13.9)	0.4 (0.249–0.723)^*^	0.5 (0.254–0.865)^*^

*COR* crude odds ratio, *AOR* adjusted odds ratio, *CI* confidence interval

^*^Statistically significant at *P* < 0.05

## Discussion

In this study, 76.7 % of the children were positive for at least one species of intestinal parasite. Similarly high prevalence of intestinal parasites was reported among school children in Tikur Wuha [9], Zarima [13] and Lake Tana Basin [20] in northwest Ethiopia. However, the prevalence of intestinal parasites observed in this study is higher compared to similar studies done in Yadot primary school [21], Jimma zone [22] and Gondar [23], in which prevalence of 26.2, 47.1 and 22.7 % were reported, respectively. Difference in environmental and socio-economic factors may account for the variation in prevalence of intestinal parasites. The high prevalence of intestinal parasites obtained in this study necessitates regular deworming of the children.

In this study, *S. mansoni* was the most prevalent intestinal parasite encountered. In Ethiopia, activities involving river water including fishing, taking bath and swimming in the river, and washing of clothes and utensils using river water are common denominators for infection with *S. mansoni* [12, 24]. In this study, it was also observed that prevalence of *S. mansoni* was significantly higher in children taking bath and washing clothes in the river. Snails of the genus *Biomphalaria* (*B. sudanica* and *B. pfeifferi*) are intermediate hosts of *S. mansoni* in Ethiopia [25, 26].

This study also revealed significantly higher prevalence of intestinal parasites among males and children aged 5–9 years. Male children usually play outdoors, and engage in outdoor activities compared to their female counterparts, which may predispose them to higher risks of IPI. Moreover, compared to females, male children are more likely to swim and take bath in the rivers, which might have contributed to the higher prevalence of *S. mansoni* observed among male children in this study. The higher prevalence of intestinal parasites in the younger children could be related to lack of awareness on transmission roots of intestinal parasites. Fentie and co-workers [20] also reported male gender as a risk factor for *S. mansoni* infection.

Fruits and vegetables may be contaminated with feco-oral parasites. In this study, consumption of raw vegetables was significantly associated with IPI. Recently, Tefera et al. [27] reported parasite contamination in more than half of the fruits and vegetables collected from local markets in Jimma town. Consumption of street-food was also significantly associated with IPIs in this study, which could be due to unsafe food handling practices by the food vendors, and unhygienic environments where the foods are sold. Moreover, children who irregularly wash hands before meal, and those drinking well and river water were significantly more infected with intestinal parasites. The significant association of lack of pure water supply and unhygienic practices with IPIs was also documented by other investigators [13, 28, 29].

In this study, significantly higher prevalence of intestinal parasites was observed among children attending public schools compared to those attending private schools. The higher prevalence of intestinal parasites among the children attending public schools could be related to lower socio-economic status of their families. Association of lower-income family with higher risk of IPI in school children has been documented elsewhere [30].

Soil-transmitted helminth and schistosome infections are usually aggregated, with few proportions of individuals harboring the highest worm burden. In this study, majority of the children had moderate-to-heavy infection with the STHs and *S. mansoni*. Pre-school and school-age children with moderate-to-heavy infection with STHs and *S. mansoni* suffer the most from health consequences of these infections [18, 31].

The major limitation of our study is that infection intensity of the STHs and *S. mansoni* was determined by examination of single stool specimen of each study participant. Moreover, a single Kato-Katz template was examined for each of the stool specimens. These might affect the accuracy of the egg count of the STHs and *S. mansoni*.

## Conclusions

This study revealed high prevalence of intestinal parasites with *S. mansoni* being the most predominant. Demographic factors and hygienic practice of the school children were associated with IPIs. Deworming of the school children following WHO guideline is urgently required. Prevention of reinfection post-deworming of the school children, and monitoring of the interventions would be vital to reduce the burden of IPIs among the school children. The health extension program and school-parent forums should emphasize on school-health.
